# AKI after pediatric cardiac surgery for congenital heart diseases–recent developments in diagnostic criteria and early diagnosis by biomarkers-

**DOI:** 10.1186/s40560-017-0242-z

**Published:** 2017-07-20

**Authors:** Yuichiro Toda, Kentaro Sugimoto

**Affiliations:** 1 Department of Anesthesiology and Intensive Care Medicine, Kawasaki Medical School, 577 Matsushima, Kurashiki-shi, Okayama 701-0192 Japan; 2 Department of Anesthesiology and Resuscitology, Okayama University Medical School, Okayama, Japan

**Keywords:** Acute kidney injury, Children, Cardiac surgery, Cardiopulmonary bypass, Biomarker, Urinary albumin, NGAL

## Abstract

**Background:**

Acute kidney injury (AKI) after cardiac surgery in children with congenital heart disease is a common complication. AKI is also associated with high morbidity and mortality. The Kidney Diseases Improving Global Outcomes (KDIGO) criteria for AKI classification are now widely used for the definition of AKI. It is noteworthy that a statement about children was added to the criteria. Many studies aimed at finding useful biomarkers are now being performed by using these criteria. Clinicians should be aware of the recent progress in understanding AKI in children.

**Main contents:**

Unlike adult patients, young age is one of the major risk factors for AKI in pediatric cardiac surgery. The mechanism of the development of AKI in children might be different from that in adults because the surgical procedure and CPB technique in pediatric patients are greatly different from those in adult patients.

There are many biomarkers for early detection of AKI, and some of them are widely used in hospitals. One of the major benefits of such biomarkers is the rapidness of expression for detecting increases in their expression levels. Neutrophil gelatinase-associated lipocalin, kidney injury molecule-1, cystatin C, and albumin have been investigated in some studies, and the usefulness of these biomarkers for detection of AKI and diagnosis of disease severity has been shown.

Although there are many interventions for preventing and treating AKI after cardiac surgery in children, there is still no specific effective treatment. Peritoneal dialysis is effective for only maintaining a negative fluid balance early after cardiac surgery. The long-term prognosis of AKI is an issue of interest. Although mortality and morbidity of AKI in the acute phase of disease remain high, the long-term condition in pediatric patients is relatively acceptable unlike in adults.

**Conclusions:**

KDIGO criteria are advocated as a diagnostic tool for common perception. Early recognition and intervention for AKI can be achieved by using several biomarkers. Further studies are needed to establish effective treatment for AKI.

## Background

Acute kidney injury (AKI) is a common comorbidity after cardiac surgery in adults [1] and children. Previously reported incidences of AKI in children after cardiac surgery have varied widely [2–4]. It has been shown that many children in whom AKI occurs have prolonged mechanical ventilation and prolonged intensive care unit stay [5, 6]. Although various criteria have been used for the diagnosis of AKI, the Kidney Disease Improving Global Outcomes (KDIGO) classification has recently been introduced as a standard diagnostic tool. Many children suffer from AKI after cardiac surgery, but there is still no specific effective treatment for AKI. The precise mechanism by which AKI develops after cardiac surgery is still not known. Early diagnostic tools such as urine and serum biomarkers have not been established, and there is still no specific treatment for preventing or curing AKI. Here, we describe transition of criteria used for diagnosis of AKI, available biomarkers, and treatment for pediatric patients after cardiac surgery.

### Mechanism of the development of AKI after cardiac surgery in children

The precise mechanism by which AKI develops after cardiac surgery is not clear because many factors are involved. The factors involved in the development of AKI include 5 large categories: preoperative, cardiopulmonary bypass (CPB), postoperative, inflammatory, and neuroendocrine factors [7]. Since kidney function in newborns is extremely limited, younger age as a risk factor for AKI is one of the differences from adults. Many adults with heart diseases have vascular changes such as arterial sclerosis, whereas microemboli during CPB is less common in pediatric patients due to the rarity of vascular diseases. Besides, the degree of inflammatory and neuroendocrine responses is considered to be much larger in children since pediatric CPB results in extensive hemodilution. Moreover, children with congenital heart disease often have various systemic to pulmonary shunts. This may make it difficult for surgeons to maintain a bloodless surgical field, often resulting in prolonged CPB time and/or intentionally decreased CPB flow.

### AKI definition

Criteria for diagnosis of AKI such as AKIN [8] and RIFLE [9] criteria have recently been proposed. The RIFLE criteria have also been modified to a pediatric version [10] using Schwartz formulae [11]. In 2012, the Kidney Disease Improving Global Outcomes (KDIGO) classification was advocated as AKI definitions in adults and children [12]. The main difference from pediatric RIFLE is the degree of creatinine change as a diagnostic tool, and an optional statement is added for pediatric AKI in stage 3 (Table 1). There is no designation about which formula should be used to calculate estimated glomerular filtration rate (GFR). However, it has been validated in a pediatric population, and higher stages have been shown to be associated with poor prognosis [13, 14].

**Table 1:** Staging of AKI (KDIGO)

Stage	Serum creatinine	Urine output
1	1.5–1.9 times baseline or ≥0.3 mg/dl (≥26.5 μmol/l) increase	<0.5 ml/kg/h for 6–12 h
2	2.0–2.9 times baseline	<0.5 ml/kg/h for ≥12 h
3	3.0 times baseline or increase in serum creatinine to ≥4.0 mg/dl (≥353.6 μmol/l) or initiation of renal replacement therapy or in patients <18 years, decrease in eGFR to <35 ml/min per 1.73 m^2^	<0.3 ml/kg/h for ≥24 h or anuria for ≥12 h

Reference [12]

### Epidemiology and risk factors

In children who underwent cardiac surgery for congenital heart diseases, the reported incidences of AKI by the pRIFLE criteria ranged from 20 to 64.6% [2, 15–17]. The reported incidences of AKI by the KDIGO classification ranged from 29 to 86% [3, 4, 18]. Possible reasons for the difference in incidences are differences in patient characteristics (age, heart disease, cardiac status), surgeon’s skill, CPB technique, management of anesthesia, and postoperative care practices. As shown in Table 2, there appears to be a higher incidence of AKI in a younger age group, whereas adults of advanced age have a higher risk of AKI.

**Table 2:** Incidence of AKI and risk factors

Author, year	*n*	Population, data source	Age (median or mean)	criteria	Incidence (%)	Risk factors
Hazle 2013 [18]	49	Single-center, prospective biomarker study	2 months	KDIGO	86	NA
Lex 2014 [3]	1489	Single-center, prospective study	1 year	KDIGO	29	NA
Meersch 2014 [17]	51	Single-center, prospective biomarker study	2 years	pRIFLE	24	NA
Gil-Ruiz Gil-Esparza 2014 [2]	409	Single-center, retrospective study	12 months	pRIFLE	26.2	Age, duration CPB, RACHS
Ruf 2015 [16]	59	Single-center, prospective spectroscopy study	2 months	pRIFLE	48	Age, univentricular anatomy
Sugimoto 2016 [15]	376	Single-center, prospective biomarker study	18 months	pRIFLE	64.6	Age, duration CPB
Park 2017 [4]	220	Single-center, retrospective study	6 months	KDIGO	41.8	Age, hemoglobin

There are many risk factors of AKI after pediatric cardiac surgery for congenital heart diseases: low body weight, young age, cyanosis, previous cardiac surgical procedure, risk adjustment in congenital heart surgery––version 1 (RACHS-1) score, univentricular anatomy, preoperative pulmonary hypertension and congestive heart failure, preoperative inotrope and captopril use, preoperative PICU admission, preoperative mechanical ventilation, calendar year (era), and study site [19]. Ruf et al. reasonably showed that low blood pressure in the first 24 h postoperatively was a risk factor [16]. There have been many studies with no detailed hemodynamic data including blood pressure as factors determining kidney perfusion. The study by Ruf et al. re-highlighted the importance of hemodynamics for the risk of AKI.

### Biomarkers

Diagnosis and severity of AKI are determined by serum creatinine and urine output. However, serum creatinine and urine output are not timely markers. The usefulness of neutrophil gelatinase-associated lipocalin (NGAL), kidney injury molecule-1 (KIM-1), cystatin-C, liver type fatty acid-binding protein (L-FABP), and interleukin (IL)-18 as markers has been shown in many studies. NGAL is the most promising marker for detecting AKI at an early phase of disease. Proteomic analysis showed that NGAL was one of the most highly induced proteins in injured distal nephron segments after ischemic or nephrotoxic AKI in animal models [20–22]. In urine that was harvested 2 h after initiation of CPB in pediatric cardiac surgery, it was found that urinary NGAL was significantly increased in children with AKI defined by serum creatinine, and the area under the curve (AUC) for detecting AKI was as high as 0.90–0.99 [23–26]. Unfortunately, measurement of new biomarkers such as NGAL, KIM-1, cystatin-C, L-FABP, and IL-18 are still expensive for them to be used as routine measurements. Furthermore, measurements must be done outside the hospital and it usually takes a few days to obtain results.

**Fig. 1:**
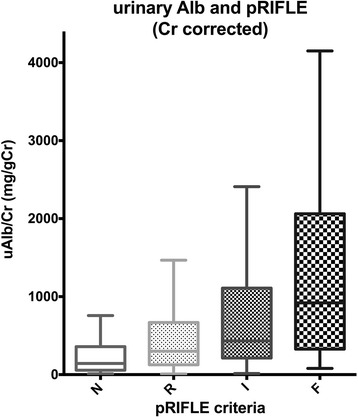
Comparison of urinary albumin in each pRIFLE category. Urinary albumin corrected by urinary creatinine. The graph shows a step-by-step increase in urinary albumin by the pRIFLE criteria. Categories in pRIFLE criteria: *N* normal, *R* risk, *I* injury, and *F* failure. Reference [15]

Urine albumin is an old but promising biomarker in this field, and several studies have reaffirmed its importance. Urine albumin can be measured in a general hospital at low cost and the results are promptly available. Generally, in the normal kidney, a small amount of serum albumin goes through the glomerular filter, and almost all of the albumin in the tubule is reabsorbed. Concomitant occurrence of an increase in albumin leakage from the glomerulus and a decrease in albumin reabsorption in the tubule results in albuminuria. It has been reported as additional mechanism that the albumin gene was induced at the renal cortex [27]. AKI can be detected earlier by urinary albumin than by serum creatinine because albumin expression occurs as early as that of NGAL or KIM-1. The diagnostic utility of urine albumin for prediction of AKI after pediatric cardiac surgery is shown in Fig. 1. The AUC for detecting AKI by urine albumin ranges 0.57 to 0.76 [15, 28–30]. These differences are justified by the wide variation of the normal range for urine albumin even in healthy individuals. The normal value of urine albumin in children varies widely depending on age [31, 32], gender [33], weight [34], and race [35]. It was shown that urine albumin corrected by urine creatinine in infants was three times higher than that in adolescents in healthy children [36]. Although a large cohort study in Europe showed that there was no difference in urine albumin levels among all age groups, urine albumin corrected by urine creatinine was higher in a younger age group [31]. There is the same problem for other biomarkers, even NGAL (which is the most extensively investigated biomarker) [37, 38]. In healthy children, approximately 50% of urine protein, mostly Tamm-Horsfall protein (uromodulin), is excreted from the tubular epithelium. Tubular proteinuria is non-reabsorption of freely filtered low-molecular-weight proteins. Albuminuria is one of major glomerular proteinuria across the glomerular capillary wall [39].

When a kidney has been damaged, urine NGAL is mainly induced from the tubule, and urine albumin is mainly induced from glomerulus. The mechanism of the development of AKI after cardiac surgery is multifactorial. The participation of both tubular and glomerular damage is one of important mechanisms for development of AKI. In order to detect AKI early and precisely, the combination of multiple AKI biomarkers should be used. Cystatin C is not bound to plasma proteins and is freely filtered by the glomerulus. Cystatin C is reabsorbed and degraded in the renal proximal tubule by the endocytic receptor megalin [40]. Unlike creatinine, cystatin C is not secreted into urine by the tubule, and its appearance in urine therefore indicates its filtration at the glomerulus and reduced uptake by the damaged proximal tubules [41]. The appearance of urinary cystatin C reflects a decrease in GFR. The feasibility of using a combination of cystatin C (functional biomarker) and NGAL (tubular biomarker) after pediatric cardiac surgery has been reported [42]. In that study, the use of combination of NGAL and cystatin C improved diagnostic precision after cardiac surgery in children.

### Management and treatment

There is still no effective specific therapy for AKI after pediatric cardiac surgery. From a physiological point of view, a reasonable intervention is maintenance of appropriate circulation and avoidance of nephrotoxic agents. In the following, issues about peritoneal dialysis (PD), aminophylline, and hydroxyethyl starch (HES) after pediatric cardiac surgery are described.

### PD

Pediatric cardiac surgery causes electrolyte disorder, acidosis, and fluid overload that are refractory to standard postoperative care. When infants or neonates are in condition of AKI, starting peritoneal dialysis (PD) is recommended. Several studies have shown that initiation of PD or placement of a PD catheter before the development of a serious condition is effective after pediatric cardiac surgery. PD after pediatric cardiac surgery has several benefits compared with continuous renal replacement therapy (CRRT) using vascular access. PD does not require anti-coagulant agents, which sometimes complicate postoperative hemostasis immediately after surgery, and does not require vascular access, which it is often difficult to obtain in small children. Kwiatkowski et al. showed the effectiveness of PD catheter placement in 42 children in a retrospective case-matched study. PD catheter placement during congenital heart surgery (PDC+ group) resulted in a significantly higher percentage of children with a negative fluid balance on postoperative days 1 and 2, shorter time to negative fluid balance, earlier extubation, improved inotrope scores, and fewer electrolyte imbalances requiring correction [43]. On the other hand, Ryerson et al. showed no effectiveness of prophylactic peritoneal dialysis catheter placement in 22 neonates in a randomized controlled trial. There were no differences between their PDC+ group and PDC− group in mean time to first postoperative negative fluid balance, time to achieve lactate ≤2 mmol/L, maximum vasoactive-inotrope scores on postoperative days 2 to 5, time to sternal closure, time to first extubation, modified clinical outcome score, and hospital length of stay [44]. Sanchez-de-Toledo et al. showed the effectiveness of early initiation of renal replacement therapy (RRT) after pediatric heart surgery in 480 patients in a single-center retrospective study. RRT techniques were used in 32 patients (6.6%), with 25 patients (78%) receiving peritoneal dialysis (PD) and 7 patients (22%) receiving continuous RRT (CRRT). Patients who received PD within the first 24 postoperative hours had lower mortality than did those in whom PD was initiated later [4/16 (25%) vs. 4/9 (44.4%)] [45]. Sasser et al. showed the effectiveness of prophylactic peritoneal dialysis following cardiopulmonary bypass in 52 children in a prospective before-and-after nonrandomized cohort study. Median net fluid balance was more negative in the prophylactic PD group at 24 h (−24 vs. +18 mL/kg) and at 48 h (−88 vs. −46 mL/kg). The prophylactic PD group had less fluid intake and lower inotrope score at 24 h and earlier sternal closure [46]. With regard to the timing for discontinuation of PD, Riley et al. showed in a prospective randomized controlled study that PD continuation for a further 24 h was not effective in 20 infants under 90 days old. Although the group with PD continuation for a further 24 h had lower mean urine output, median levels of AKI biomarkers did not differ significantly between the groups [47]. Prophylactic peritoneal dialysis catheter placement and early initiation of PD may be effective, but more prospective randomized studies are needed.

### Aminophylline

Theophylline is recommended as class 2B. A single dose of theophylline may be given in neonates with severe perinatal asphyxia, who are at high risk of AKI in the KDIGO AKI practice guidelines [12]. Both theophylline and aminophylline are xanthine derivatives and have a strong diuretic effect. Axelrod et al. showed no effectiveness of aminophylline infusion in a single-center, double-blinded, placebo-controlled, randomized clinical trial for 72 children after pediatric cardiac surgery. Aminophylline was administered every 6 h for 72 h in the ICU. There was no significant difference between the incidences of AKI in the aminophylline group and placebo group [48]. Onder et al. showed that the intraoperative usage of aminophylline was more effective than furosemide in reversal of oliguria in the early postoperative period in a single-center, historical control, retrospective cohort study for 200 children after pediatric cardiac surgery. There was no significant difference between the incidences of AKI during a period of 48 h in the aminophylline group and furosemide group [49]. Their study indicates that the effectiveness of aminophylline is limited.

### HES

Hydroxyethyl starch (HES) has been used for cheaper and safer volume replacement fluids than albumin solution. However, one of the problems for HES infusion is the possibility of development of renal injury by interstitial proliferation, macrophage infiltraton, and tubular damage [50]. In 7000 adult ICU patients, the use of 6% HES 130/0.4 was associated with a higher incidence of the requirement for RRT [51]. However, a correction was recently made for adverse events in the journal, and an editor in BMJ was concerned the reliability of data [52, 53]. There is a limited information on HES infusion in pediatric patients after cardiac surgery. Van Der Linden et al. showed that the effectiveness of 6% HES 130/0.4 was equal to that of 5% albumin for kidney injury in 61 cardiac surgery children in a randomized, controlled, parallel-group, double-blinded trial. HES and 5% albumin were used for intraoperative volume replacement including priming of the extracorporeal circuitry. Urinary renal biomarkers (α1-microglobulin, β-N-acetylglucosaminidase, NGAL, and albumin) increased in all patients after surgery but without significant differences between the HES group and 5% albumin group [54]. Van Der Linden et al. also showed in a retrospective propensity-matched study that the effectiveness of 6% HES 130/0.4 was equal to that of 4% albumin for kidney injury in 1495 cardiac surgery children with CPB [55]. In that study, there was no difference between the groups in the incidence of postoperative renal failure requiring renal replacement therapy. Akkucuk et al. showed that HES usage as CPB priming solution did not have a negative effect on renal function compared with Ringer’s lactate in 24 cardiac surgery children with CPB in a prospective, randomized study. From CPB initiation to 48 h postoperatively, there were no differences between the groups in cystatin C, β2-microglobulin, fractional excretion of sodium (FENa), urine albumin/creatinine ratio, creatinine clearance, and urine output [56].

### Prognosis

Many studies have shown that the development of AKI after pediatric cardiac surgery was associated with poor short-term prognosis including ICU stay, duration of mechanical ventilation, and mortality. Recently, the long-term prognosis has been highlighted. In an adult population after cardiac surgery, it was shown that AKI was associated with poor long-term prognosis including chronic kidney disease and mortality [57–59]. In a pediatric population, Cooper et al. showed that AKI-positive patients and AKI-negative patients had similar normal assessments of kidney function by eGFR, similar proteinuria, and similar blood pressure at long-term follow-up (mean duration of 7 years) in 51 children in a single-center, cross-sectional study [60]. Watkins et al. reported that pRIFLE stage F was significantly associated with higher mortality at long-term follow-up (mean duration of 4 years) in a single-center, retrospective study with 718 children [61]. Mel et al. showed that long-term renal prognosis in survivors was good in 76 children in whom postoperative AKI developed and who were managed with PD in a single-center, cohort study at long-term follow-up (3.5–10.5 years). Of the 76 children included in that study, 35 died during the immediate postoperative period, 15 died during the interim of nonrenal causes, and 26 were alive at the time of follow-up evaluation [62]. There is a possibility that pediatric patients with AKI after cardiac surgery have a different prognosis from that for adult patients with AKI after cardiac surgery. More data from a prospective study are needed.

## Conclusions

Frequent incidences of AKI after pediatric cardiac surgery are recognized. Morbidity and mortality rates in patients with AKI are high for both children and adults. The KDIGO criteria are useful for diagnosing AKI, even in children. Biomarkers for AKI including NGAL, cystatin C, and albumin have become available, and they will enable early and timely intervention. However, only PD seems to be an effective treatment at the current stage. The long-term outcome in children with AKI might be different from that in adults.

## Abbreviations

AKI: Acute kidney injury
AKIN: Acute kidney injury network
AUC: Area under curve
CPB: Cardiopulmonary bypass
CRRT: Continuous renal replacement therapy
eCCl: Estimated creatinine clearance
eGFR: Estimated glomerular filtration rate
HES: Hydroxyethyl starch
KDIGO: Kidney Diseases Improving Global Outcomes
KIM-1: Kidney injury molecule - 1
L-FABP: Liver fatty acid-binding protein
LMW: Low molecular weight
NGAL: Neutrophil gelatinase-associated lipocalin
PD: Peritoneal dialysis
RIFLE: Risk, injury, failure, loss, end stage kidney disease
UO: Urine output
